# Maternal white blood cell count cannot identify the presence of microbial invasion of the amniotic cavity or intra-amniotic inflammation in women with preterm prelabor rupture of membranes

**DOI:** 10.1371/journal.pone.0189394

**Published:** 2017-12-12

**Authors:** Ivana Musilova, Lenka Pliskova, Romana Gerychova, Petr Janku, Ondrej Simetka, Petr Matlak, Bo Jacobsson, Marian Kacerovsky

**Affiliations:** 1 Department of Obstetrics and Gynecology, Charles University Faculty of Medicine in Hradec Kralove, University Hospital Hradec Kralove, Hradec Kralove, Czech Republic; 2 Institute of Clinical Biochemistry and Diagnostics, University Hospital Hradec Kralove, Hradec Kralove, Czech Republic; 3 Department of Obstetrics and Gynecology, University Hospital Brno, Faculty of Medicine Masaryk University, Brno, Czech Republic; 4 Department of Gynecology and Obstetrics, University Hospital Ostrava, Ostrava, Czech Republic; 5 Department of Obstetrics and Gynecology, Sahlgrenska Academy, Gothenburg University, Gothenburg, Sweden; 6 Domain of Health Data and Digitalization, Norwegian Institute of Public Health, Oslo, Norway; 7 Biomedical Research Center, University Hospital Hradec Kralove, Hradec Kralove, Czech Republic; Johns Hopkins University, UNITED STATES

## Abstract

**Objective:**

The main aim of this study was to determine the relationship between the maternal white blood cell (WBC) count at the time of hospital admission in pregnancies complicated by preterm prelabor rupture of membranes (PPROM) and the presence of microbial invasion of the amniotic cavity (MIAC) and/or intra-amniotic inflammation (IAI). The second aim was to test WBC diagnostic indices with respect to the presence of MIAC and/or IAI.

**Methods:**

Four hundred and seventy-nine women with singleton pregnancies complicated by PPROM, between February 2012 and June 2017, were included in this study. Maternal blood and amniotic fluid samples were collected at the time of admission. Maternal WBC count was assessed. Amniotic fluid interleukin-6 (IL-6) concentration was measured using a point-of-care test, and IAI was characterized by an IL-6 concentration of ≥ 745 pg/mL. MIAC was diagnosed based on a positive polymerase chain reaction result for the *Ureaplasma* species, *Mycoplasma hominis*, and/or *Chlamydia trachomatis* and/or for the 16S rRNA gene.

**Results:**

Women with MIAC or IAI had higher WBC counts than those without (with MIAC: median, 12.8 × 10^9^/L vs. without MIAC: median, 11.9 × 10^9^/L; *p* = 0.0006; with IAI: median, 13.7 × 10^9^/L vs. without IAI: median, 11.9 × 10^9^/L; *p* < 0.0001). When the women were divided into four subgroups based on the presence of MIAC and/or IAI, the women with both MIAC and IAI had a higher WBC count than those with either IAI or MIAC alone, and those without MIAC and IAI [both MIAC and IAI: median, 14.0 × 10^9^/L; IAI alone: 12.1 × 10^9^/L (*p* = 0.03); MIAC alone: 12.1 × 10^9^/L (*p* = 0.0001); and without MIAC and IAI: median, 11.8 × 10^9^/L (*p* < 0.0001)]. No differences in the WBC counts were found among the women with IAI alone, MIAC alone, and without MIAC and IAI.

**Conclusion:**

The women with both MIAC and IAI had a higher maternal WBC count at the time of hospital admission than the remaining women with PPROM. The maternal WBC count at the time of admission showed poor diagnostic indices for the identification of the presence of both MIAC and IAI. Maternal WBC count at the time of admission cannot serve as a non-invasive screening tool for identifying these complications in women with PPROM.

## Introduction

Preterm prelabor rupture of membranes (PPROM), characterized by the rupture of the fetal membranes, with leakage of amniotic fluid, before spontaneous onset of regular uterine contractions prior to 37 weeks of gestation, has been considered a difficult, serious, and controversial perinatal complication since many years [1, 2]. Characterization of PPROM pregnancies differs based on their causality and pathophysiology: i) infection and inflammation in the choriodecidual space and amniotic cavity; ii) bleeding associated with placental abruption; and iii) non-infectious premature aging of the fetal membranes [1–6]. Regardless of the PPROM etiology and pathophysiology, PPROM might jeopardize the fetus by premature birth and its consequences [7]. In addition, PPROM might threaten both the fetus and the mother because of the presence of microbial invasion of the amniotic cavity (MIAC) and intra-amniotic inflammation (IAI), which may lead to the development of histological and even clinical chorioamnionitis [8–10].

At this stage, evaluation of amniotic fluid samples alone, obtained via transabdominal amniocentesis, might present precise information on the intra-amniotic environment. The feasibility of transabdominal amniocentesis in PPROM dramatically changes over time (the success rate has increased from 49% to 96%), mainly owing to the progress in ultrasound technology [11, 12]. In addition, this procedure can be considered safe for both mothers and fetuses [12]. Nonetheless, the majority of obstetricians are reluctant to perform amniocentesis in women with PPROM owing to its invasiveness and the relative technical difficulty associated with the scenario, which involves a very low volume of residual amniotic fluid. Therefore, non-invasive markers for the identification of intra-amniotic complications associated with PPROM are still needed.

Various markers from non-invasively obtained body fluids have been suggested over the decades; however, the evaluation of maternal white blood cell (WBC) count in the 1990s could be considered an important pioneering step in the process of a non-invasive biomarker-seeking process [11, 13–15]. Currently, the WBC count has not been taken into consideration as a very useful marker for infection-related and inflammatory intra-amniotic PPROM complications for at least two reasons: i) a wide range of normal maternal WBC count during pregnancy [16] and ii) the studies with the presence of acute histological chorioamnionitis, clinical chorioamnionitis, and neonatal infection as the outcomes have reported that maternal WBC counts were poor diagnostic indices for these outcomes [10, 11, 13–15, 17–24]. Despite these data, the evaluation of maternal WBC count is still a part of the clinical protocol in women with PPROM in a number of countries worldwide.

Since women with PPROM prior to the 34^th^ completed gestational week are typically treated expectantly, the latency (the interval between rupture of the membranes and delivery) might be long. Therefore, the presence of histological chorioamnionitis and the development of neonatal infection as outcomes, cannot appropriately describe the actual intra-amniotic environment at the time of admission to hospital of women with PPROM. For this reason, MIAC and IAI appear to be ideal outcomes, since the sampling of both the maternal and intra-amniotic environment might be performed at the same time. Nevertheless, there is a paucity of information to whether and how the presence of MIAC and/or IAI affects the maternal WBC count at the time of admission of women with PPROM.

Therefore, the main aim of this study was to determine the relationship between maternal WBC counts on admission to hospital and the presence of MIAC and IAI in women with PPROM. The second aim of this study was to assess the association between the maternal WBC count and amniotic fluid interleukin (IL)-6 concentrations at the time of admission. The final aim was to characterize the maternal WBC count at the time of admission in four subgroups of women with PPROM subdivided on the basis of the presence and/or absence of MIAC and/or IAI.

## Materials and methods

This prospective cohort study was conducted between February 2012 and June 2017. Women admitted to the Department of Obstetrics and Gynecology, University Hospital in Hradec Kralove, Czech Republic, were recruited if they had pregnancies complicated by PPROM between gestational ages 24+0 and 36+6 weeks. Only women aged at least 18 years with a singleton pregnancy were eligible for the study. Women with any medical complications (e.g., hypertension, preeclampsia, diabetes mellitus, and thyroid disease), fetal growth restriction, gross vaginal bleeding, signs of fetal hypoxia, and structural malformations or chromosomal abnormalities of the fetus were excluded from the study. Gestational age was established for all pregnancies based on the first trimester ultrasound results.

PPROM was defined as the leakage of amniotic fluid prior to the onset of labor and was diagnosed visually via a sterile speculum examination to confirm the pooling of amniotic fluid in the vagina. In case of clinical doubt, PPROM was confirmed by the presence of insulin-like growth factor-binding protein (ACTIM PROM test; Medix Biochemica, Kauniainen, Finland) in the vaginal fluid.

Women with PPROM at less than 34 weeks of gestation were treated using tocolytics for 48 hours, antibiotics, and corticosteroids to accelerate lung maturation. Those with PPROM beyond 34 weeks of gestation were treated only with antibiotics. The women with PPROM included in this study were managed with two different approaches. Between February 2012 and December 2013, transabdominal amniocenteses were performed only for research purposes, and the results from amniotic fluid analyses were not used for clinical management. During this period women with PPROM were treated actively (except those at < 28 gestational weeks). Labor was induced, or an elective cesarean section was performed no later than 72 hours after the rupture of the membranes, depending on the gestational age of the pregnancy, fetal status, and maternal serum C-reactive protein concentrations. Since January 2014, the performance of transabdominal amniocentesis procedures and collection of information on the presence of MIAC and/or IAI have become a routine part of the clinical management of women with PPROM at our department. Thus, women with PPROM admitted between January 2014 and July 2017 were managed differently. Women with both MIAC and IAI beyond the 28th gestational week were managed actively (labor was induced, or an elective cesarean section was performed after finalizing corticosteroid treatment within 72 hours of membrane rupture for pregnancies before 34 weeks of gestational age and within 24 hours of membrane rupture for those beyond 34 weeks). The remaining women with PPROM were managed expectantly. Since maternal and intra-amniotic samples were obtained simultaneously during the hospital admission, the differences in management protocols over the study period could not affect the results of this study.

This study’s protocol was approved by the Ethics Committee of the University Hospital of Hradec Kralove, Czech Republic (March 19, 2008; No. 200804 SO1P, which was renewed in July 2014; decision No. 201407 S14P), and written informed consent was obtained from all the participants.

Amniotic, cervical, crevicular, and vaginal fluid samples from this cohort of women have been used in our previously published studies [8, 25–45]. One hundred and ninety-two and 287 women from this study have taken part in our previous studies on the associations between maternal serum C-reactive protein concentrations and MIAC and/or histological chorioamnionitis and MIAC and/or IAI, respectively [37, 45].

### Maternal blood and amniotic fluid sampling

For all women, the maternal blood and amniotic fluid samples were collected at the time of hospital admission (maternal blood first, followed by amniotic fluid) prior to the administration of corticosteroids, antibiotics, or tocolytics. Maternal blood samples were obtained via venipuncture of the cubital vein and were sent to the laboratory for the assessment of the WBC count immediately following sampling. Ultrasound-guided transabdominal amniocentesis was performed, and ~5 mL of amniotic fluid was aspirated. A total of 100 μL of non-centrifuged amniotic fluid was used for the bedside assessment of interleukin (IL)-6 concentrations, and one tube with non-centrifuged amniotic fluid was transported to the laboratory for DNA isolation, detection of the *Ureaplasma* species, *Mycoplasma hominis (M*. *hominis)*, and *Chlamydia trachomatis (C*. *trachomatis)* using polymerase chain reaction (PCR), and 16S rRNA gene sequencing.

### Amniotic fluid IL-6 concentrations

The amniotic fluid IL-6 concentrations were assessed using the Milenia QuickLine IL-6 lateral flow immunoassay on the Milenia^®^ POC Scan Reader (Milenia Biotec, GmbH, Giessen, Germany). The measurement range was 50–10,000 pg/mL. The intra-assay and inter-assay coefficients of variation were 12.1% and 15.5%, respectively [26].

### Detection of the *Ureaplasma* species, *M*. *hominis*, and *C*. *trachomatis*

DNA was isolated from the amniotic fluid using a QIAamp DNA Mini Kit (Qiagen, Hilden, Germany) according to the manufacturer’s instructions (using the protocol for isolation of bacterial DNA from biological fluids). Real-time PCR was conducted on a Rotor-Gene 6000 instrument (Qiagen) using the commercial kit AmpliSens^®^*C*. *trachomatis/Ureaplasma/M*. *hominis*-FRT (Federal State Institution of Science, Central Research Institute of Epidemiology, Moscow, Russia) to detect the DNA of the *Ureaplasma* species, *M*. *hominis*, and *C*. *trachomatis* using the same PCR tube. As a control, we included a PCR for β-actin, a housekeeping gene, to examine for the presence of PCR inhibitors [46]. The amount of target DNA in the sample was measured based on the threshold cycle (Ct) value [47]. The Ct value is the intersection between an amplification curve and a threshold line. It is a measurement method used to determine the concentration of the target DNA in the PCR. Under ideal conditions, the amount of target amplicon increases at a rate of one log_10_ every 3.32 cycles, which means that decreasing the microbial load of the *Ureaplasma* species in the amniotic fluid results in higher Ct values [47].

### Detection of other bacteria in the amniotic fluid

Bacterial DNA was identified by PCR targeting the 16S rRNA gene using the following primers: 5′-CCAGACTCCTACGGGAGGCAG-3′ (V3 region) and 5′-ACATTTCACAACACGAGCTGACGA-3′ (V6 region) [48, 49]. The PCR products from 16S rRNA were purified and used in sequencing the PCR reactions with the abovementioned primers and the BigDye Terminator kit, v3.1 (Thermo Fisher Scientific, Waltham, MA, USA). The bacteria were then typed using the sequences obtained in the BLAST^®^ and SepsiTest^™^ BLAST databases.

### Diagnosis of MIAC

MIAC was diagnosed using a non-culture-based approach. MIAC was defined as a positive PCR result for the *Ureaplasma* species, *M*. *hominis*, and/or *C*. *trachomatis* or positivity of the 16S rRNA gene in the amniotic fluid.

### Diagnosis of IAI

IAI in pregnancies with PPROM was defined as bedside amniotic fluid IL-6 concentrations of ≥ 745 pg/mL [50, 51].

### Statistical analyses

The demographic and clinical characteristics of women were compared using the nonparametric Kruskal-Wallis test for continuous variables and were presented as median values (ranges). Categorical variables were compared using the chi-square test and were presented as numbers (%). The maternal WBC counts were compared using either the Mann-Whitney *U* test or Kruskal-Wallis test with *post hoc* Dunn’s analysis, as appropriate, and presented as median values [interquartile ranges (IQRs)]. Spearman’s partial correlation was used to adjust the significance of the results for potential confounders (gestational age at sampling, parity, latency from PPROM to amniocentesis, and smoking history). To identify an association between maternal WBC count and microbial loads of the *Ureaplasma* species and amniotic fluid IL-6 concentrations, Spearman’s correlations were used. Differences were considered significant at *p* < 0.05. All *p*-values were obtained in two-sided tests, and all statistical analyses were performed using the GraphPad Prism version 6.0 software for Mac OS X (GraphPad Software, San Diego, CA, USA) or the SPSS version 19.0 statistical package for Mac OS X (SPSS Inc., Chicago, IL, USA).

## Results

### Demographic and clinical characteristics of the study population

**Fig 1:**
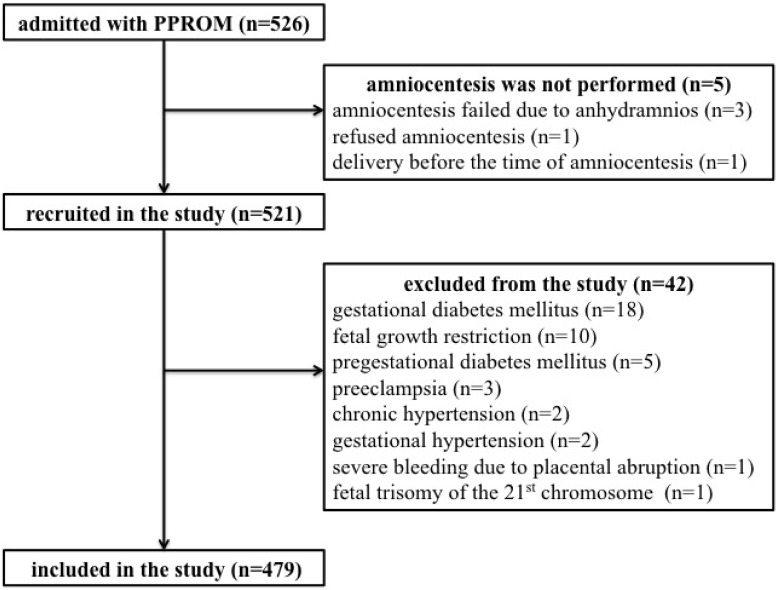
Flow diagram describing the selection of women for the study.

A total of 526 women with singleton pregnancy complicated by PPROM were admitted during the study period, although only 479 women were included in the analyses. The flow diagram describing the selection of women is shown in Fig 1.

The presence of MIAC and IAI was identified in 27% (130/479) and 21% (99/479) of the women, respectively. When the women were divided into four subgroups based on the presence and absence of MIAC and IAI, the presence of both MIAC and IAI was found in 14% (68/479); the presence of IAI alone in 7% (31/479); the presence of MIAC alone in 13% (62/479); and the presence of neither MIAC nor IAI was found in 66% (318/479) of the women. The demographic and clinical characteristics of the women with PPROM with respect to the presence of MIAC and/or IAI are shown in Table 1. The most common bacteria found in the amniotic fluid were the *Ureaplasma* species, which were identified in 19% (89/479) of the women. Polymicrobial findings in the amniotic fluid were observed in 4% (19/479) of the women. All microbial findings are shown in Table 2.

**Table 1:** Demographic and clinical characteristics of women with PPROM stratified according to the presence and absence of MIAC and/or IAI.

Characteristic	With MIAC and IAI (n = 68)	With IAI alone (n = 31)	With MIAC alone (n = 62)	Without MIAC and IAI (n = 318)	*p* value
Maternal age [years, median (range)]	31 (17–42)	30 (21–38)	31 (18–42)	31 (18–43)	0.90
Primiparous [number (%)]	25 (37%)	17 (55%)	27 (44%)	169 (53%)	0.06
Pre-pregnancy body mass index [kg/m^2^, median (range)]	22.5 (17.4–38.2)	24.2 (18.3–38.6)	22.1 (16.0–35.1)	22.7 (16.4–57.4)	0.09
Smoking history [number (%)]	25 (37%)	3 (10%)	11 (18%)	31 (10%)	**0.0001**
Gestational age at admission [weeks, median (range)]	31+3 (24+0–36+6)	31+3 (24+0–36+6)	34+3 (25+3–36+6)	34+0 (24+0–36+6)	**< 0.0001**
Gestational age at delivery [weeks, median (range)]	31+4 (24+0–36+6)	32+5 (25+0–37+0)	34+6 (26+6–37+0)	34+2 (24+0–36+6)	**< 0.0001**
Interval from PPROM to amniocentesis [hours, median (range)]	7 (1–575)	4 (2–432)	4 (1–206)	5 (1–600)	0.06
Latency from amniocentesis to delivery [hours, median (range)]	45 (3–624)	71 (4–1392)	37 (4–390)	39 (2–768)	0.05
Amniotic fluid IL-6 concentration [pg/mL, median (range)]	10000 (747–10000)	1363 (751–10000)	186 (50–673)	183 (50–729)	**< 0.0001**
CRP concentration at admission [mg/L, median (range)]	12.6 (0–113.0)	6.3 (1.0–59.3)	4.3 (0–28.9)	4.9 (0–47.5)	**< 0.0001**
Administration of antibiotics [number (%)]	67 (99%)	30 (97%)	62 (100%)	313 (98%)	0.66
Administration of corticosteroids [number (%)]	54 (79%)	21 (68%)	37 (60%)	198 (62%)	**0.03**
Spontaneous vaginal delivery [number (%)]	44 (65%)	21 (68%)	50 (81%)	216 (68%)	0.19
Cesarean delivery [number (%)]	24 (35%)	10 (32%)	11 (18%)	98 (31%)	0.14
Forceps delivery [number (%)]	0 (0%)	0 (0%)	1 (2%)	4 (1%)	0.71
Birth weight [grams, median (range)]	1620 (650–3540)	1945 (660–3320)	2300 (780–3250)	2245 (680–3670)	**< 0.0001**
Apgar score < 7; 5 minutes [number (%)]	4 (6%)	2 (6%)	0 (0%)	8 (3%)	0.14
Apgar score < 7; 10 minutes [number (%)]	1 (2%)	1 (3%)	0 (0%)	4 (1%)	0.62

Abbreviations: PPROM: preterm prelabor rupture of membranes; MIAC: microbial invasion of the amniotic cavity; IAI: intra-amniotic inflammation; IL: interleukin; CRP: C-reactive protein

Continuous variables are compared using the nonparametric Kruskal-Wallis test. Categorical variables are compared using the chi-square test. Statistically significant results are marked in bold. Continuous variables are presented as medians (ranges) and categorical variables as numbers (%).

**Table 2:** The bacterial species identified in the amniotic fluid of women with PPROM.

Women with MIAC and IAI (n = 68)	Women with MIAC alone (n = 62)
*Ureaplasma* species (33)	*Ureaplasma* species (37)
*Ureaplasma* species + *Mycoplasma hominis* (6)	*Ureaplasma* species + *Chlamydia trachomatis* (5)
*Ureaplasma* species + *Chlamydia trachomatis* (3)	*Ureaplasma* species + *Leptotrichia amnionii* (1)
*Ureaplasma* species + *Lactobacillus* species (1)	*Mycoplasma hominis* (6)
*Ureaplasma* species + *Sneathia sanguinegens* (1)	*Chlamydia trachomatis* (2)
*Ureaplasma* species + *Enterococcus faecium* (1)	*Bifidobacterium* species (1)
*Chlamydia trachomatis* (1)	*Gardnerella vaginalis* (1)
*Chlamydia trachomatis* + *Leptotrichia amnionii* (1)	*Lactobacillus gasseri* (2)
*Haemophilus influenza* (3)	*Propionibacterium acnes* (2)
*Fusobacterium nucleatum* (2)	*Staphylococcus haemolyticus* (1)
*Leptotrichia amnionii* (1)	*Staphylococcus warneri* (1)
*Parvimonas micra* 1x	*Streptococcus intermedius* 1x
*Peptococcus* species 1x	*Streptococcus pneumoniae* 1x
*Peptoniphilus* species 1x	*Streptococcus salivarius* 1x
*Propionibacterium acnes* 1x	
*Propionibacterium* species 1x	
*Sneathia sanguinegens* 1x	
*Staphylococcus hominis* 1x	
*Streptococcus agalactiae* 3x	
*Streptococcus condemnatus* 1x	
*Streptococcus intermedius* 1x	
*Streptococcus* species 1x	
Bacteria non-identifiable by sequencing 2x	

Abbreviations:

PPROM: preterm prelabor rupture of membranes; MIAC: microbial invasion of the amniotic cavity; IAI: intra-amniotic inflammation

Women with PPROM included in this study were treated with two different management strategies (an active management between January 2012 and December 2013 and an individualized management based on the presence of both MIAC and IAI between January 2014 and December 2017). The active management was associated with a lower gestational age at delivery (medians: 33+4 weeks vs. 34+2 weeks; *p* = 0.002), birth weight (medians: 2020 g vs. 2240 g; *p =* 0.01), a shorter latency (medians: 40 hours vs. 48 hours; *p* = 0.04), and a higher rate of cesarean section (60% vs. 35%; *p* = 0.004). All the women were self-reported Caucasians.

### Maternal WBC counts based on the presence of MIAC

**Fig 2:**
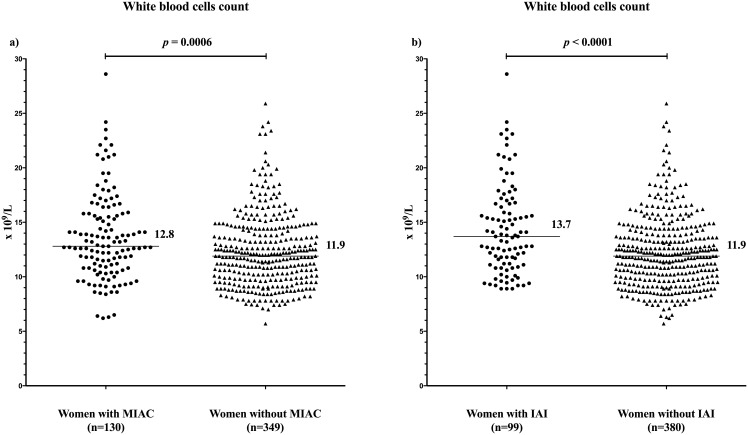
**Fig 2a.** Maternal WBC counts in the PPROM pregnancies complicated by the presence of MIAC. The women with MIAC have a higher maternal WBC count than the women without these conditions. Abbreviations: WBC, white blood cell count; PPROM, preterm prelabor rupture of membranes; MIAC, microbial invasion of the amniotic cavity. **Fig 2b.** Maternal WBC counts in the PPROM pregnancies complicated by the presence of IAI. The women with IAI have a higher maternal WBC count than the women without these conditions. Abbreviations: WBC, white blood cell count; PPROM, preterm prelabor rupture of membranes; IAI, intra-amniotic inflammation.

The women with MIAC had higher WBC counts than the women without MIAC (with MIAC: median, 12.8 × 10^9^/L, IQR: 10.8–15.8 × 10^9^ vs. without MIAC: median, 11.9 × 10^9^/L, IQR: 9.8–14.4 × 10^9^; *p* = 0.0006; Fig 2a) in the crude analysis, as well as after adjustments for gestational age at sampling, smoking, parity, and interval from PPROM to amniocentesis (*p* = 0.02). No differences in the maternal WBC count between the women with genital mycoplasmas (*Ureaplasma* species and/or *M*. *hominis*) and women with the other bacteria in the amniotic fluid were found (with genital mycoplasmas: median, 12.7 × 10^9^/L, IQR: 10.5–15.8 × 10^9^ vs. with the other bacteria: median, 13.1 × 10^9^/L, IQR: 11.2–15.8 × 10^9^; *p* = 0.59). A weak correlation between the microbial burden of the *Ureaplasma* species (characterized by Ct values) in the amniotic fluid and maternal WBC count was identified (rho = -0.33, *p =* 0.002).

### Maternal WBC counts based on the presence of IAI

Women with IAI had higher WBC counts than the women without IAI (with IAI: median, 13.7 × 10^9^/L, IQR: 11.2–16.7 × 10^9^ vs. without IAI: median, 11.9 × 10^9^/L, IQR: 9.8–13.9 × 10^9^; *p* < 0.0001; Fig 2b) in the crude analysis and in the adjusted analysis for gestational age at sampling, smoking history, parity, and interval from PPROM to amniocentesis (*p* < 0.0001). A weak correlation between the maternal WBC count and amniotic fluid IL-6 concentrations was found (rho = 0.24; *p* < 0.0001).

### Maternal WBC counts based on the presence of MIAC and/or IAI

**Fig 3:**
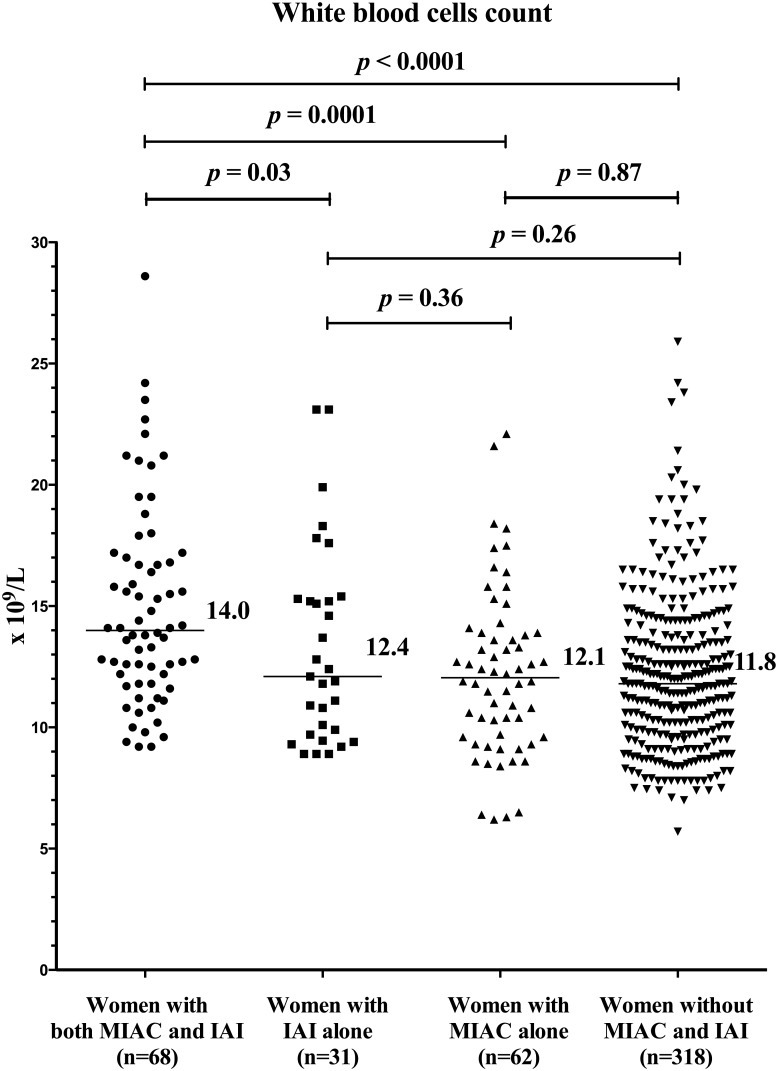
Maternal serum WBC counts in the PPROM pregnancies complicated by the presence of MIAC and/or IAI. The women with both MIAC and IAI have a higher WBC count than the women with IAI alone, MIAC alone, and neither MIAC nor IAI. Abbreviations: WBC, white blood cell count; PPROM, preterm prelabor rupture of membranes; MIAC, microbial invasion of the amniotic cavity; IAI, intra-amniotic inflammation.

When the women were divided into four subgroups according to the presence of MIAC and/or IAI, differences in WBC counts on admission were identified (women with both MIAC and IAI: median, 14.0 × 10^9^/L, IQR: 11.9–17.0 × 10^9^; women with IAI alone: median, 12.1 × 10^9^/L, IQR: 9.7–15.3 × 10^9^; women with MIAC alone: median, 12.1 × 10^9^/L, IQR: 9.6–13.9 × 10^9^; and women without both MIAC and IAI: median, 11.8 × 10^9^/L, IQR: 9.8–13.9 × 10^9^; *p* < 0.0001; Fig 3) in the crude analysis, as well as after adjustments for gestational age at sampling, smoking history, parity, and interval from PPROM to amniocentesis (*p* < 0.0001). The women with both MIAC and IAI had higher WBC counts than the women with IAI alone (*p* = 0.03), women with MIAC alone (*p* = 0.0001), and women without MIAC and IAI (*p* < 0.0001). No differences in the WBC counts were identified among the women with IAI alone or MIAC alone and without both MIAC and IAI (IAI alone vs. MIAC alone: *p* = 0.36; IAI alone vs. without both MIAC and IAI: *p* = 0.26; and MIAC alone vs. without both MIAC and IAI: *p* = 0.87).

**Fig 4:**
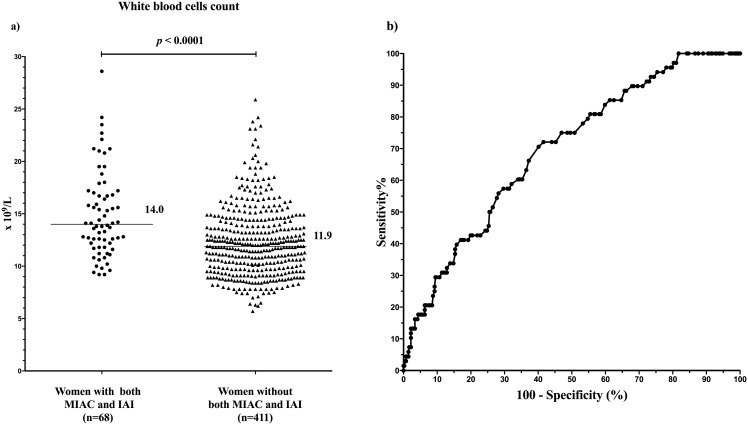
Maternal WBC counts stratified according to the presence or absence of both MIAC and IAI. A receiver-operating characteristic curve for the presence of both MIAC and IAI is shown (the area under the curve is 0.70 for the WBC cutoff value of >14.0 × 10^9^/L; *p* < 0.0001). Abbreviations: WBC, white blood cell count; MIAC, microbial invasion of the amniotic cavity; IAI, intra-amniotic inflammation.

The WBC count cutoff value of 14.0 × 10^9^/L was found to be the most effective in identifying both MIAC and IAI, with a sensitivity of 50% [34/68; 95% confidence interval (CI), 38–62%], specificity of 75% (306/411; 95% CI, 70–78%), positive predictive value of 25% (34/139; 95% CI, 18–32%), negative predictive value of 90% (306/340; 95% CI, 86–93%), positive likelihood ratio of 2.0 (95% CI, 1.5–2.6), negative likelihood ratio of 0.7 (95% CI, 0.5–0.9), and area under the receiver-operating characteristic curve of 0.70 (95% CI, 0.63–0.76; *p* < 0.0001; Fig 4).

## Discussion

The assessment of WBC counts from blood samples is one of the most common approaches in evaluating the systemic inflammatory response and its intensity in non-pregnant women. During pregnancy, the maternal WBC count assessment has limited value owing to a broader range of reference values present across the trimesters than during non-pregnancy periods [16]. Nevertheless, there is still a gap in the knowledge with regard to whether and how the presence of MIAC and/or IAI affects the maternal WBC count in PPROM.

In this study, we attempted to bridge this gap in knowledge by providing the following key findings: i) the presence of MIAC was associated with a higher maternal WBC count at the time of admission; ii) no differences in the maternal WBC count between the women with genital mycoplasmas and the other bacteria in the amniotic fluid was found; iii) the microbial load of the *Ureaplasma* species weakly correlated with the maternal WBC count; iv) the presence of IAI correlated with a higher maternal WBC count at the time of admission to hospital; v) the maternal WBC count weakly correlated with amniotic fluid IL-6 concentrations; vi) the women with both MIAC and IAI had a higher maternal WBC count at the time of admission than the women with MIAC alone, IAI alone, and neither MIAC nor IAI; and vii) the maternal WBC count obtained at the time of admission had poorer diagnostic indices for identifying the presence of both MIAC and IAI.

The presence of MIAC has been shown to complicate about one third of PPROM pregnancies and to be associated with higher intra-amniotic, maternal inflammatory responses at the time of admission, as well as the fetal inflammatory response at the time of delivery [8, 19, 34, 52, 53]. In 1996, Yoon et al. published their study conducted on 90 women with PPROM where they showed that women with MIAC had higher maternal WBC counts at the time of admission to hospital [19]. Our findings are in accordance with the results of their study. When taken together, we confirmed that the presence of MIAC is associated with a higher maternal inflammatory response at the time of admission, even when measured by the WBC count. However, the small differences in the medians of maternal WBC counts between women with and without MIAC prevent the WBC count from being a useful clinical marker in predicting MIAC.

MIAC is a very heterogeneous condition complicated by the invasion of different microbes in the amniotic fluid and by their different microbial loads. Genital mycoplasmas are the most common and account for up to 75% of all the microbial species identified in the amniotic fluid [34, 52, 54] from women with PPROM [34, 52, 54]. There are conflicting results regarding the intra-amniotic inflammatory response elicited by genital mycoplasmas [54–56]. To date, it is not completely clear whether there may be differences in intra-amniotic inflammatory responses elicited by genital mycoplasmas compared to those elicited by other bacteria [54–56]. Therefore, it would be of interest to better discriminate whether the presence of genital mycoplasmas in the amniotic fluid is associated with different maternal inflammatory responses as measured by the maternal WBC count as compared with the presence of other bacteria. In this study, we found no difference. However, this finding is not in accordance with that of a previous study by Oh et al., where the authors found a higher maternal inflammatory response in the subgroup with genital mycoplasmas [54]. The possible explanation for these conflicting results might be due to the fact that the intra-amniotic and maternal inflammatory responses to genital mycoplasmas have been shown to be dependent on microbial load [47, 57, 58]. Accordingly, we found a correlation between the microbial load of the *Ureaplasma* species and maternal WBC count. This finding is in line with that of our previous study showing the correlation between the maternal serum C-reactive protein concentrations and the microbial loads of the *Ureaplasma* species [47].

A plethora of researchers have evaluated the association between maternal WBC count and histological chorioamnionitis [11, 13–15, 17–24, 59]. However, there is limited information regarding the relationship between maternal WBC count and IAI [60]. Ferrazzi et al. found in cohort of 23 women that those with IAI had a higher maternal WBC count than those without IAI [60]. This finding has been confirmed in our study performed on 479 women with PPROM. Moreover, we have shown that the maternal inflammatory response, characterized by the maternal WBC count, reflects the intensity of the IAI response as determined by the amniotic fluid IL-6 concentration. This finding is very important from a clinical standpoint, since it shows that even a subclinical inflammatory intra-amniotic complication might affect the systemic maternal compartment.

Recently, the subdivision of women with PPROM into four subgroups based on the presence and absence of MIAC and/or IAI has been proposed by Romero et al. [61]. This group subdivision enables a more precise characterization of the intra-amniotic environment at the time of amniotic fluid sampling. The subgroup of women with both MIAC and IAI (microbial-induced IAI) has been shown to have the most intensive intra-amniotic and cervical inflammatory responses [32, 34, 61]. In a recent study from our group, the maternal inflammatory response, determined by the maternal serum C-reactive protein concentration, has been found to be the strongest in the subgroup of women affected by both MIAC and IAI [45]. The observation from the current study conducted on an approximately two-fold larger cohort of women, is in line with this finding; women with both MIAC and IAI had a higher maternal WBC count than the women in the remaining subgroups. This finding is clinically relevant as it suggests that this subgroup of PPROM differs from the others. Therefore, this specific subgroup of PPROM should receive particular attention and be treated more cautiously.

There is a solid body of evidence indicating that smoking is associated with an elevated WBC count [62, 63]. Interestingly, we found the highest number of smokers in the subgroup of women with the highest maternal WBC count, i.e. in women with both MIAC and IAI. The same subgroup of women had the longest interval from PPROM to amniocentesis. This means that the intra-amniotic environment of this subgroup of women was exposed to the cervical and vaginal microbiota longer than the remaining women. This longer exposure might have contributed to the development of MIAC and IAI. Therefore, smoking and the interval from PPROM to amniocentesis should be considered as potential confounders and the results should be interpreted, accordingly.

Over the years, different maternal WBC count cutoff values have been proposed to predict MIAC, histological or clinical chorioamnionitis, and neonatal infection [11, 13–15, 17–24, 59]. Although maternal WBC count can easily be evaluated between the time of admission and delivery, there are differences among previous studies with regard to the temporal relationship between the maternal blood WBC count sampling and outcomes. Using the time of admission as a time-point for the sampling, the suggested WBC count cutoff values to predict MIAC and histological or clinical chorioamnionitis have ranged from 10 x 10^9^/L to 16 x 10^9^/L [11, 14, 19, 23, 24]. However, none of these values have good predictive indices. To test whether maternal WBC count obtained at the time of admission could be a potential predictor of PPROM complicated by both MIAC and IAI, we identified the maternal WBC count cutoff value 14.0 × 10^9^/L as ideal. This cutoff value is almost in the middle of the aforementioned proposed WBC count range. This cutoff value showed a good negative predictive value, but its positive predictive value and likelihood ratio were poor, as such, the latter prevents this WBC count cutoff value from being used in the clinical setting.

A major strength of this study is the fact that it contains a relatively large cohort of women with a clearly defined, specific phenotype of spontaneous preterm delivery (PPROM). Second, a transabdominal amniocentesis was successfully performed to obtain a sample of amniotic fluid in 99% of the women admitted to hospital. Third, the maternal serum and amniotic fluid samples were obtained simultaneously at the time of admission. Therefore, the maternal systemic inflammatory response, measured by the maternal WBC count, could be used to indicate the actual status of the intra-amniotic compartment. Fourth, the microbial load of the amniotic fluid *Ureaplasma* species was assessed. Lastly, the results were adjusted for gestational age at sampling, parity, the interval from PPROM and amniocentesis, and smoking, since these variables might have been as potential confounding factors.

Several limitations of this study should also be mentioned. First, since the study focused only on the association between the single assessment of the maternal WBC count at admission and the current intra-amniotic status, it did not contain any information on the temporal trend of the maternal WBC count during the latency period, nor on the association between the maternal WBC count and histological chorioamnionitis. Second, the upper detection limit of amniotic fluid IL-6 assessment was 10,000 pg/mL. Since 39 out of 479 women reached this upper detection limit, this might have affected the results regarding the correlation between the maternal serum WBC count and amniotic fluid IL-6 concentrations. Next, an extensive non-culture approach (combination of specific PCR for the *Ureaplasma* species, *M*. *hominis*, and *C*. *trachomatis* and non-specific PCR for 16S rRNA gene) was used to evaluate MIAC in this study; however, underemphasizing of the MIAC negative group cannot be ruled out as the culture data is missing [52]. Lastly, the amniotic fluid WBC count was not evaluated, since this was not part of our clinical management of PPROM.

In conclusion, women with PPROM complicated by both MIAC and IAI had a higher maternal WBC count than the women without these conditions or than women exhibiting either MIAC or IAI alone. The maternal WBC count at the time of hospital admission showed poor diagnostic indices for the identification of the presence of both MIAC and IAI. Thus, the maternal WBC count cannot serve as a non-invasive screening tool in identifying these complications in women with PPROM.

## Supporting information

S1 Dataset (XLS)
